# Assessment of heterosis based on parental genetic distance estimated with SSR and SNP markers in upland cotton (*Gossypium hirsutum* L.)

**DOI:** 10.1186/s12864-021-07431-6

**Published:** 2021-02-18

**Authors:** Xiaoli Geng, Yujie Qu, Yinhua Jia, Shoupu He, Zhaoe Pan, Liru Wang, Xiongming Du

**Affiliations:** 1 State Key Laboratory of Cotton Biology, Institute of Cotton Research, Chinese Academy of Agricultural Sciences, Anyang, 455000 China; 2 Zhengzhou Research Base, State Key Laboratory of Cotton Biology, Zhengzhou University, Zhengzhou, 450001 China

**Keywords:** Upland cotton, SSR, SNP, Genetic distance, Heterosis

## Abstract

**Background:**

Heterosis has been extensively utilized in different crops and made a significant contribution to global food security. Genetic distance (GD) is one of the valuable criteria for selecting parents in hybrid breeding. The objectives of this study were to estimate the GD between parents using both simple sequence repeat (SSR) markers and single nucleotide polymorphism (SNP) markers and to investigate the efficiency of the prediction of hybrid performance based on GD. The experiment comprised of four male parents, 282 female parents and 1128 F_1_, derived from NCII mating scheme. The hybrids, their parents and two check cultivars were evaluated for two years. Performance of F_1_, mid-parent heterosis (MPH), and best parent heterosis (BPH) were evaluated for ten agronomic and fiber quality traits, including plant height, boll weight, boll number, lint percentage, fiber length, fiber strength, fiber uniformity, fiber elongation ratio, micronaire, and spinning consistent index.

**Results:**

Heterosis was observed in all hybrids and, the traits like plant height, boll number, boll weight and lint percentage exhibited higher heterosis than the fiber quality traits. Correlations were significant between parental and F_1_ performances. The F_1_ performances between three hybrid sets (Elite×Elite, Exotic×Elite, and Historic×Elite) showed significant differences in eight traits, including boll number, lint percentage, fiber length, fiber strength, fiber uniformity, fiber elongation ratio, micronaire, and spinning consistent index. The correlation of the GD assessed by both SSR and SNP markers was significantly positive. The cluster analysis based on GD results estimated using SNP showed that all the female parents divided into five groups and the F_1_ performance between these five groups showed significant differences in four traits, including lint percentage, micronaire, fiber strength, and fiber elongation ratio. The correlation between GD and F_1_ performance, MPH and BPH were significant for lint percentage and micronaire.

**Conclusions:**

Our results suggested that GD between parents could be helpful in heterosis prediction for certain traits. This study reveals that molecular marker analysis can serve as a basis for assigning germplasm into heterotic groups and to provide guidelines for parental selection in hybrid cotton breeding.

**Supplementary Information:**

The online version contains supplementary material available at 10.1186/s12864-021-07431-6.

## Background

Cotton is the most important natural fiber crop in the world and one of the cultivated allotetraploid Upland cotton (*Gossypium hirsutum* L.) fulfills about 95% of the output of global cotton production [1]. Heterosis or hybrid vigor is used to describe the phenomenon that the F_1_ hybrids present superior performance than parents [2]. Utilization of heterosis in cotton has significantly contributed to the yield and fiber quality [3]. The development of hybrid cotton involves the proper selection of parents and the identification of superior heterotic combinations. Screening a large number of parental lines and selecting appropriate parents for crossing and evaluating them in multiple locations is laborious, costly, and time-consuming. Various methods have been used to predict the hybrid performance depending on the types of hybrids (single cross or three-way cross) and traits which including parental performance, mid-parent value and the general combining ability [4–7].

With the aim of saving resources, the genetic distance (GD) inferred from molecular markers has been suggested as a promising tool for hybrid performance prediction and recognition of heterotic groups [8–10]. Recently, several reports concerning maize, rice, wheat have suggested the possibility of using the molecular markers, such as simple sequence repeat (SSR) and single nucleotide polymorphism (SNP), to select parental materials for heterosis crosses [6, 11–13]. According to these literatures, there is a regression of either hybrid performance or heterosis with increasing molecular genetic distance. These studies showed the potential of GD in the prediction of hybrid performance for important traits.

Several studies in cotton have used molecular markers such as restriction fragment length polymorphism (RFLP), randomly amplified polymorphic DNA (RAPD), or SSR to estimate GD among parents and use their values to predict the hybrid performance, heterosis or specific combining ability (SCA) [14–16]. But these studies were based on a rather small set of parental lines and the marker density was very low. Because the cotton genome has tremendously redundant sequences, therefore the assessment of cotton GD requires high-density molecular markers.

The present study used 286 Upland cotton accessions to construct 1128 hybrids according to North Carolina (NC II) mating design and investigated ten agronomic and fiber quality traits and heterosis. We used both SSR and SNP markers to estimate the GD between parents. We further analyzed the relationship between GD and heterosis, and assessed the feasibility of the use of SSR and SNP based genetic distances in predicting the hybrid performance and heterosis.

## Results

### Genetic distance and clustering analysis for the population

In this study, both SSR and SNP markers were used to investigate the genetic distance **(**GD) between parents. A total of 198 polymorphic SSR markers were distributed on 26 chromosomes. There were 557 polymorphic alleles in 286 parents ranged from one to ten alleles per marker with an average of 2.81. For the SNP markers, with a missing rate greater than 30% and minor allele frequency (MAF) less than 5% were eliminated and a total of 76,654 SNPs were obtained. These SNPs distributed on 26 chromosomes and varied in density at different chromosomes and locations (Fig. S1).

**Fig. 1:**
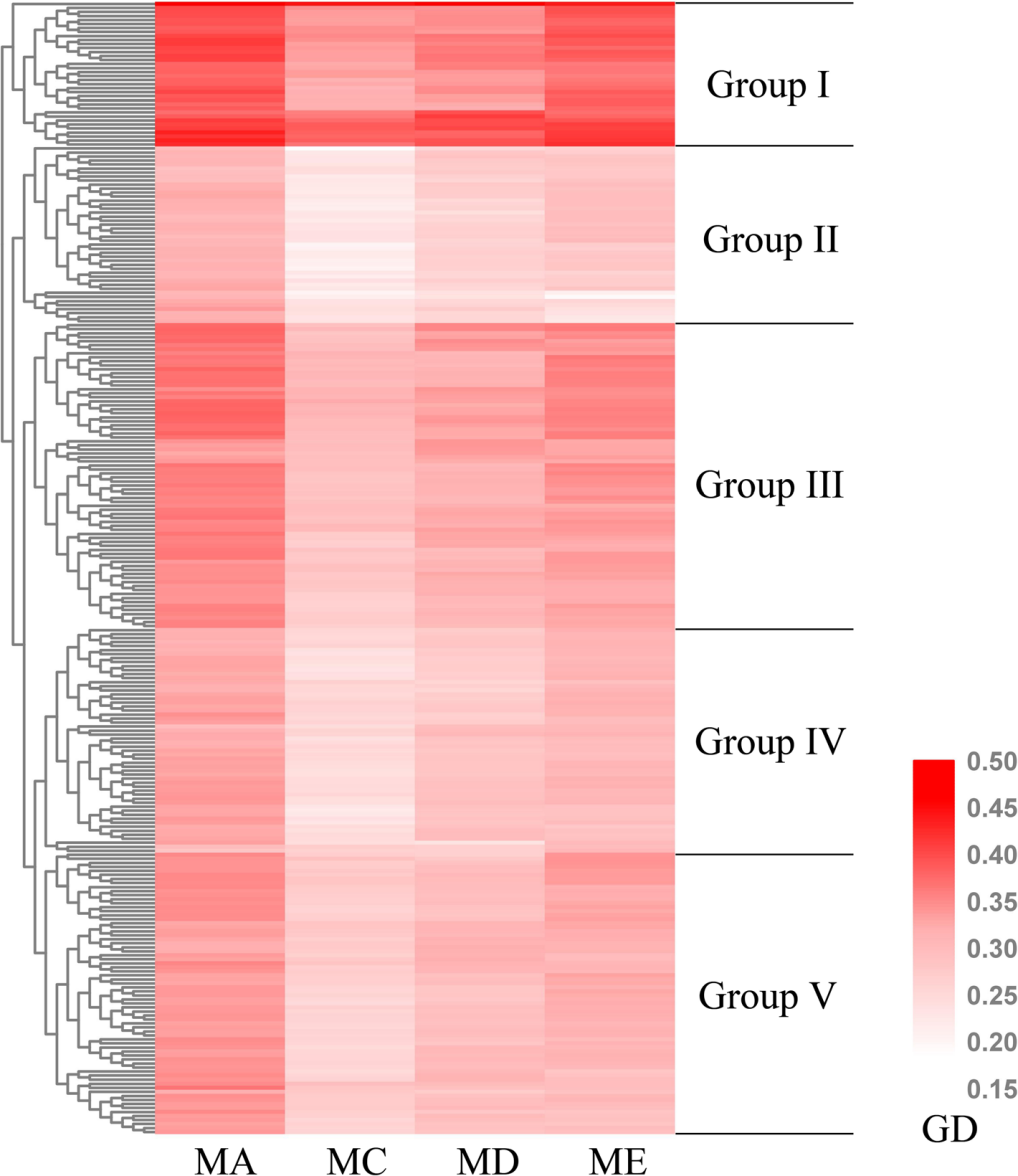
Clustering of 1128 F_1_s into five groups using genetic distance based on SNP markers

The GD between the parents calculated based on SSR markers showed that the GD between four male parents (Zhong7886, A971, 4133, and SGK9708) and 282 female parents varied from 0.139 to 0.387, with an average of 0.279 (Table 1, Table S1). The F_1_ population which crossed from four male parents was named as population A (Zhong7886), C (A971), D (4133), and E (SGK9708) according to their male parents. The mean value of GD assessed by SSR markers in each F_1_ populations was E > C > D > A. The GD between parents based on SNP markers showed that the GD varied from 0.137 to 0.375, with an average of 0.242 (Table 1, Table S1). The mean value of GD assessed by SNP markers in each F_1_ populations was A > E > D > C. The correlation of the GD assessed by SSR and SNP markers was significantly positive (0.264 ≤ *r* ≤ 0.375, *P* < 0.01). Furthermore, 1128 F_1_ hybrids clustered into five groups based on GD assessed through SNP markers and named as group I, II, III, IV and V, having 144, 176, 304, 224 and 280 F_1_, respectively (Fig. 1). From the clustering results by SSR, all the F_1_ hybrids could be clustered into three groups, which contained 536, 468, and 124 F_1_ hybrids and names as group 1, 2 and 3, respectively (Fig. S2). But the clustering results by SSR was not perfectly match the clustering results by SNP. Although we could find that Group 1 in SSR clustering result included the majority crosses which clustered as Group I and Group III by SNP, Group 2 in SSR clustering result was consisted by crosses which clustered as Group III, Group IV, and Group V by SNP, and Group 3 in SSR clustering result included the majority crosses which clustered as Group II by SNP. Moreover, because the number of the SNP marker was significantly larger than SSR marker, so we decided to use the clustering results by SNP to do the further analysis.

**Table 1:** Summary of genetic distance estimated between parents using both SSR and SNP markers

Male parent	SSR marker			SNP marker			Correlation
	Min	Max	Average	Min	Max	Average	
MA	0.149	0.348	0.264	0.219	0.375	0.272	0.264**
MC	0.212	0.387	0.313	0.137	0.362	0.209	0.341**
MD	0.180	0.373	0.275	0.175	0.374	0.235	0.363**
ME	0.139	0.375	0.375	0.145	0.357	0.252	0.375**

*, ** Indicate significance at *P* < 0.05 and *P <* 0.01, respectively.

### Performance of F_1_ hybrids among different population groups

**Fig. 2:**
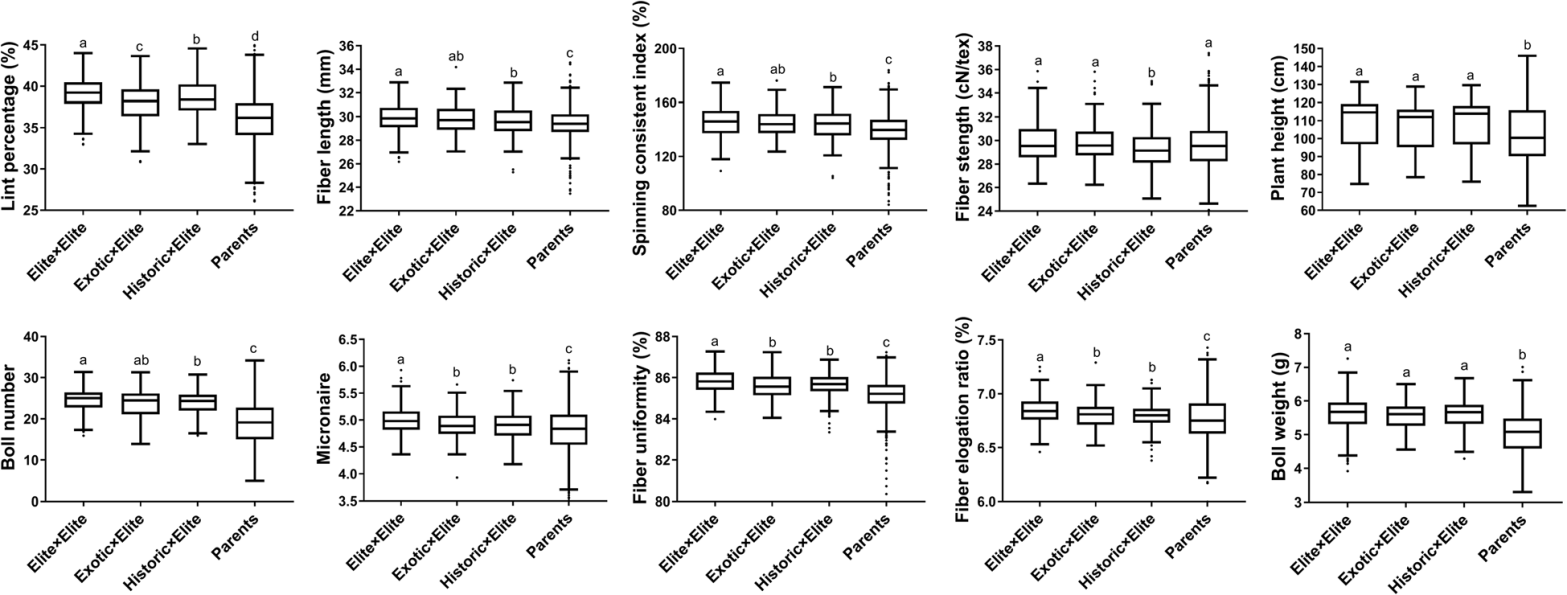
Boxplots showing the distribution of F_1_ performance for ten traits for the Elite×Elite, Exotic×Elite, and Historic×Elite hybrids and parent performances

In this study, according to the cultivated years and origins, all the 286 parents could be divided into three groups, which named Elite cultivars, Historical cultivars, and Exotic cultivars. Elite cultivars were cultivated in China after 2000, Historical cultivars were cultivated in China before 2000 and exotic cultivars were collected from other countries except of China. Therefore, this study included three different sets of cotton hybrids, termed Elite×Elite, Exotic×Elite, and Historic×Elite. The Elite×Elite hybrids showed significant lower GD than the other two hybrids sets (Fig. S3). Furthermore, we evaluated the F_1_ performance of the Elite×Elite, Exotic×Elite, and Historic×Elite hybrids and made comparisons with parent performances, and the result showed that all the F_1_ hybrid performance were significantly higher than parents in all the nine traits except of fiber strength (Fig. 2). The lint percentage (LP) decreased significantly from the Elite×Elite to Historic×Elite and Exotic×Elite hybrids. For fiber length (FL) and spinning consistent index (SCI), the mean value of Elite×Elite hybrids was significantly higher than the Historic×Elite hybrids. For fiber strength (FS), the mean value of Historic×Elite hybrids-was significantly lower than the Elite×Elite and Exotic×Elite hybrids. For boll number (BN), micronaire (MIC), fiber uniformity (FU) and fiber elongation rate (FE), the mean value of Elite×Elite hybrids was significantly higher than both the Exotic×Elite and Historic×Elite hybrids. However, no significant differences were observed for plant height (PH) and boll weight (BW) between these three hybrid sets.

**Fig. 3:**
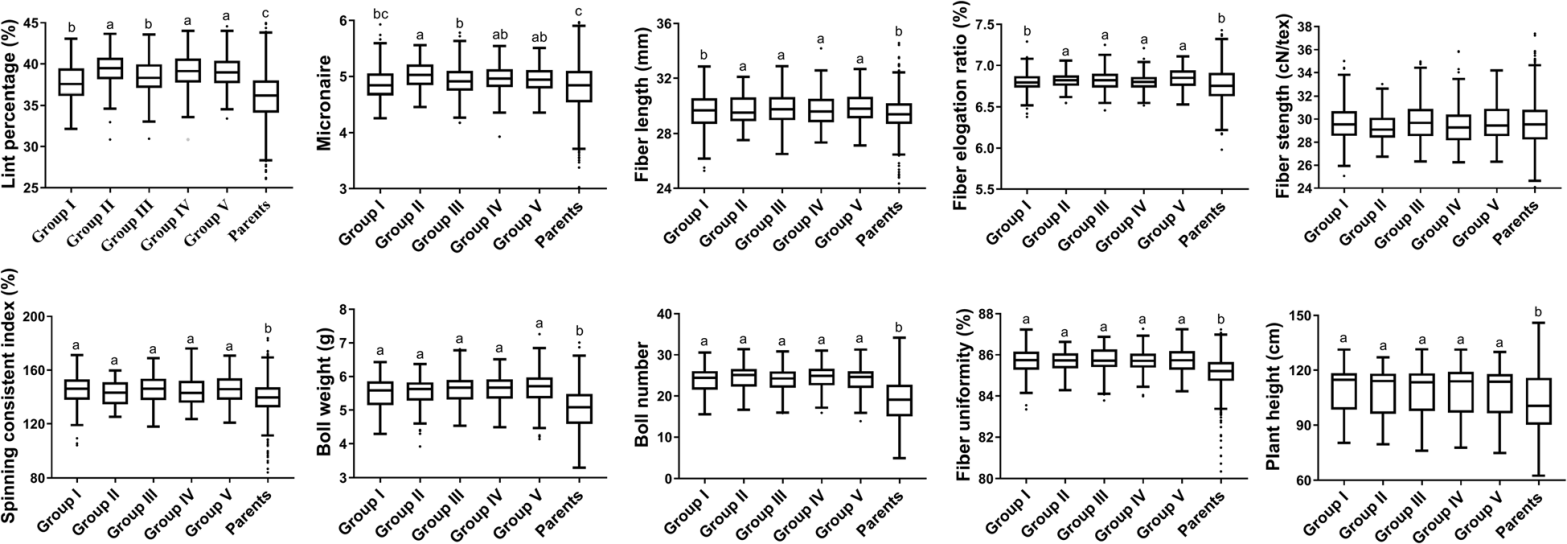
Boxplots showing the distribution of F_1_ performance for ten traits for the groups I, II, III, IV, and V and parent performances

From the above clustering result by SNP, we concluded that all 1128 hybrids could be divided into five groups according to the GD, therefore we compared the F_1_ hybrid performance of the each group and parents (Fig. 3). Firstly, seven traits showed significantly higher values in both five F_1_ groups than parents except of FL, FE, and FS. Secondly, Group II, IV, and V showed significantly higher LP than group I and III while Group II showed significantly MIC than group I and III. Furthermore, the mean values of group II, III, IV, and V for FL and FE were significantly higher than parent except of group I. For FS, there was no difference between all the F_1_ hybrids with parents. Finally, there was no significant differences among each F_1_ groups for SCI, BW, BN, FU, and PH. All these results demonstrated that different groups showed varied performances for concerning trait.

### Heterosis performance of F_1_ hybrids

**Fig. 4:**
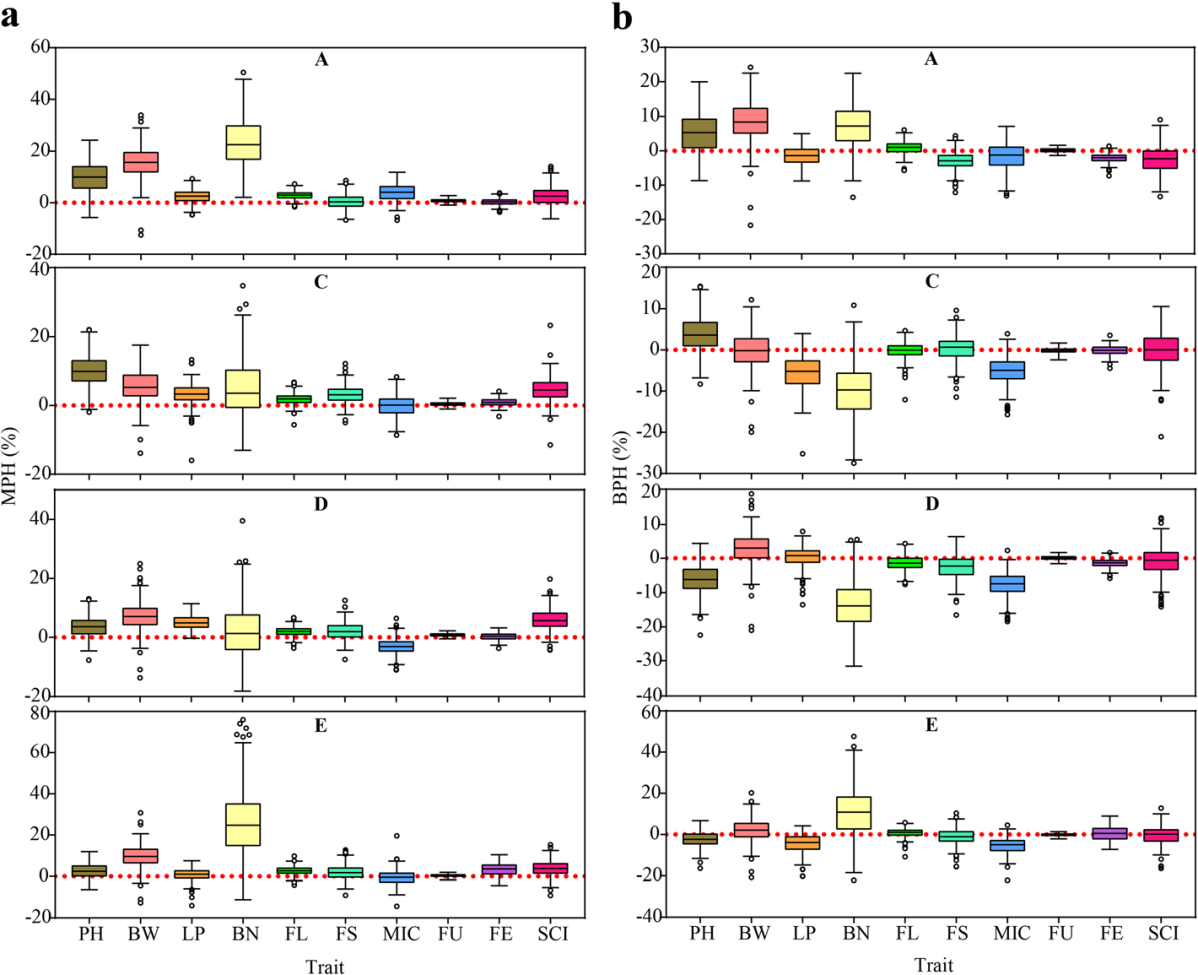
Heterosis performance of F_1_ hybrids. a: boxplots showed the mid-parent heterosis for all the analyzed traits. b: boxplots showed the better-parent heterosis for all the analyzed traits. PH: plant height, BW: boll weight, LP: lint percentage, BN: boll number, FL: fiber length, FS: fiber strength, MIC: micronaire, FU: fiber uniformity, FE: fiber elongation, SCI: spinning consistency index

We compared the mid-parent heterosis (MPH) and best-parent heterosis (BPH) of ten traits in 1128 F_1_ hybrids and the results showed that the MPH values ranged from − 18.2 to 75.9%, whereas the BPH values varied from − 31.4 to 47.7%. The mean values of MPH of the ten traits ranged from 0.09 to 14.18%, with an average of 4.36%, and the mean values of BPH ranged from − 4.85 to 3.30%, with an average of − 0.86%. Generally, the mean BPH values were lower than the MPH values for all traits, and approximately 80.9 and 41.6% of the crosses had positive MPH and BPH, respectively (Fig. 4). Among the different F_1_ populations, F_1_ population derived from the male parent A (Zhong7886) had higher MPH and BPH values than the other three F_1_ populations. As compared to yield-related traits (PH, BW, LP and BN), much less MPH and BPH were found for the fiber quality traits. Almost negligible MPH (− 1.81 to 2.76%) and BPH (− 2.38 to 1.70%) were observed for FU, suggesting that this trait was mainly controlled by additive effect.

### Correlation between parent performance, F_1_ performance and heterosis

**Fig. 5:**
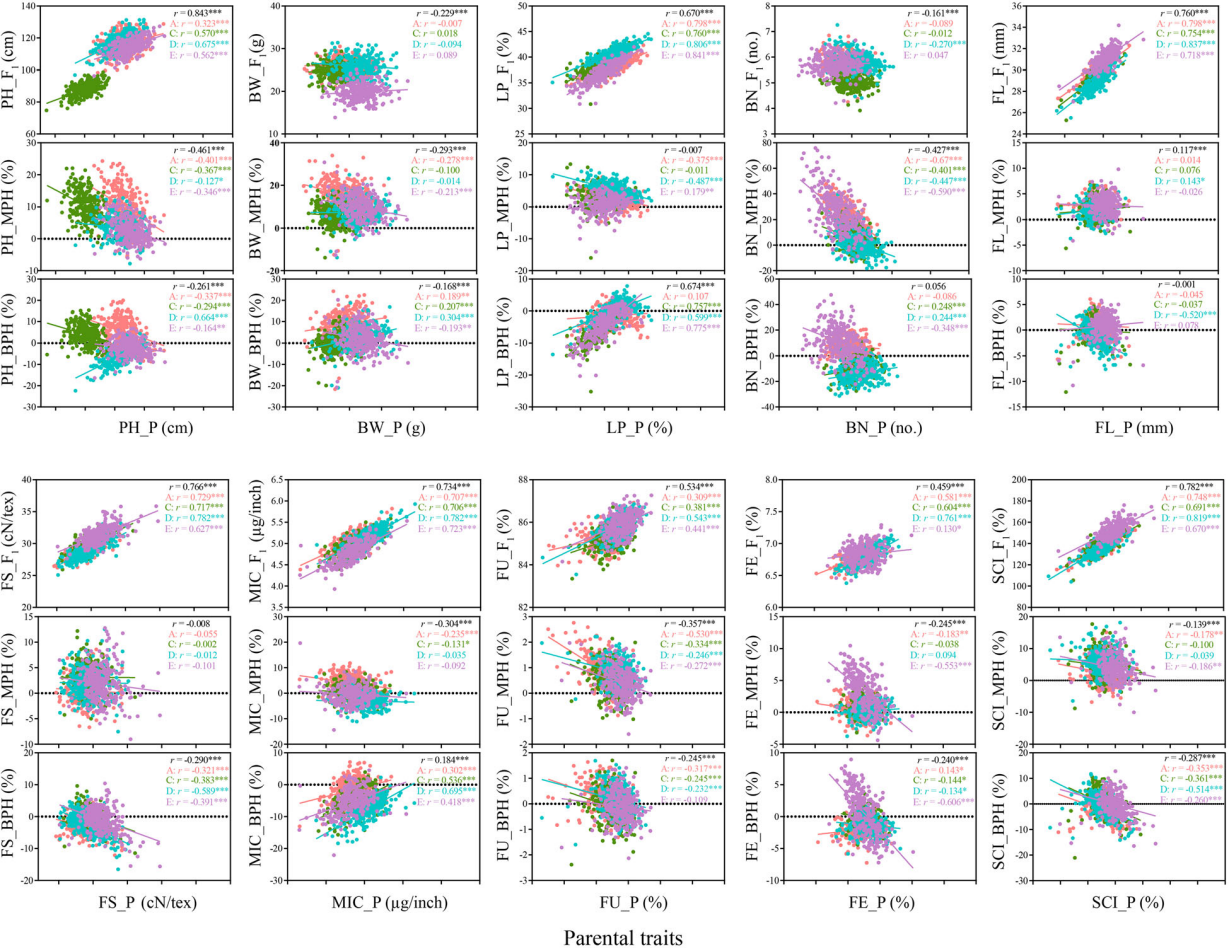
Correlation matrix between parental performance and F_1_ performance and heterosis

The correlation analysis between the performance of parents and the hybrid performance was studied to investigate the effect of the parents on the performance of the hybrids. The result showed that the correlation between parents and F_1_s performance was significantly positive (ranged from 0.459 to 0.843) in the ten traits except BW and BN. Therefore, this result suggested that genetic control of these traits was under additive genes, and the performance of parents can be used to predict the hybrid performance of these eight traits except for BW and BN (Fig. 5).

The performance of parents showed significant negative correlation with MPH of PH, BN, MIC, and FU (ranged from − 0.127 to − 0.670) in all four populations. For BW, FE and SCI, the correlation between parent performance and MPH values showed significant negative association only in population A and E. While, FL showed significant negative correlation between parents and MPH in population D. For LP, significant negative correlation was observed between parents and MPH in population A and D, but showed significant positive correlation in population E. There was no significant correlation observed between parent performance and MPH for FS (Fig. 5).

The correlation statistics between parent performance and BPH showed that only the correlations for MIC (0.184) were significantly positive in all the four populations, but for FS and SCI, the correlations were significantly negative in all the four populations, ranged from − 0.260 to − 0.589. For LP, the correlation between parents and BPH showed significant positive correlation in population C, D and E. For FU, parents and BPH showed significant negative correlation in group A, C and D. For FE, the correlation between parents and BPH showed significant positive correlation only in group A. The correlation for FL between parents and BPH showed significant negative correlation only in group D. While, PH, BW and BN have both Positive and negative correlations in the four populations (Fig. 5).

### Correlation between genetic distance and F_1_ performance

To understand the effect of genetic distance of the parents on the level of heterosis in hybrids, the correlations between genetic distance and the F_1_ performance, MPH, and BPH were calculated.

Based on the correlation between the GD of SSR markers and F_1_ performance, the GD_SSR_ was negatively correlated with BW, LP, BN, FL, MIC, and FU in at least one F_1_ population, but not significantly correlated with PH, FE and SCI (Table 2). However, GD_SSR_ was positively correlated with FS in the D population. Based on the correlation between the GD of SNP markers and F_1_s performance, GD_SNP_ was negatively correlated with LP, BN, FL, MIC and FE in at least one F_1_ population but not significantly correlated with other traits like PH, BW and FS (Table 2). However, GD_SNP_ was only positively correlated with SCI in the C population.

**Table 2:** Correlation coefficients (*r*) of genetic distance with F_1_s performance of yield and fiber quality-related traits based on SSR and SNP marker.

Trait	GD_SSR_				GD_SNP_			
	A	C	D	E	A	C	D	E
PH	-0.106	-0.006	0.031	0.057	-0.060	0.115	0.030	0.060
BW	-0.151*	-0.096	0.044	-0.026	0.044	-0.058	0.025	0.044
LP	-0.198**	-0.203**	-0.249**	-0.182**	-0.365**	-0.318**	-0.369**	-0.360**
BN	-0.025	-0.140*	-0.063	-0.204**	-0.168**	-0.128*	-0.060	-0.235**
FL	-0.043	-0.046	-0.066	-0.121*	-0.123*	-0.048	0.027	-0.122*
FS	0.069	0.056	0.137*	-0.065	0.025	0.104	0.091	0.038
MIC	-0.063	-0.204**	-0.044	-0.197**	-0.214**	-0.250**	-0.122*	-0.210**
FU	-0.054	0.012	-0.038	-0.172**	-0.046	0.069	-0.001	-0.050
FE	-0.067	-0.056	0.048	-0.023	-0.153*	-0.034	0.018	-0.105
SCI	0.027	0.078	0.083	-0.072	0.033	0.147*	0.096	0.020

GD_SSR_: genetic distance calculated based on SSR marker; GD_SNP_: genetic distance calculated based on SNP marker. A, C, D, and E indicate the F_1_ population, respectively. PH, plant height; BW, boll weight; LP, lint percentage; BN, boll number; FL, fiber length; FS, fiber strength; MIC, micronaire; FU, fiber uniformity; FE, fiber elongation rate; SCI, spinning consistency index. *, ** Indicate significance at *P* < 0.05 and *P* < 0.01, respectively.

Overall, most of the traits were negatively correlated with GD_SSR_ and GD_SNP_, and only two traits (FS and SCI) were positively correlated with GD_SSR_ and GD_SNP_ in only one population. Furthermore, GD_SNP_ had more effective power than GD_SSR_.

### Relationship between genetic distance and MPH

The correlation between GD of SSR markers and MPH showed that GD_SSR_ was negatively correlated with FL, FS, MIC, FU, and SCI in population E, but positively correlated with MPH for PH and BW in population E and D, respectively (Table 3). The correlation results between GD of SNP markers and MPH showed that the GD_SNP_ was positively correlated with the MPH of PH, BN, FS and FU in only one population and positively correlated with BW and SCI in two populations (Table 3). For the MPH of LP, the correlation was positive in the D population but negative in population E.

**Table 3:** Correlation coefficients (*r*) of genetic distance with mid-parent heterosis (MPH) of yield and fiber quality-related traits based on SSR and SNP marker.

Trait	GD_SSR_				GD_SNP_			
	A	C	D	E	A	C	D	E
PH	0.006	-0.002	0.132	0.225**	-0.019	0.061	0.095	0.147*
BW	0.065	0.031	0.118*	0.094	0.058	0.024	0.140*	0.157**
LP	0.088	-0.073	-0.027	-0.043	0.101	-0.058	0.147*	-0.125*
BN	0.106	0.002	0.067	0.097	0.146*	0.072	0.096	0.116
FL	0.006	0.044	0.082	-0.161**	0.023	0.039	0.061	-0.040
FS	0.023	0.041	0.079	-0.146*	0.058	0.121*	0.057	0.091
MIC	0.054	-0.031	-0.007	-0.170**	-0.010	-0.074	0.004	-0.073
FU	0.066	0.105	0.035	-0.135*	0.097	0.118*	0.071	0.063
FE	0.004	-0.007	0.060	0.055	-0.013	0.095	0.039	0.009
SCI	0.050	0.077	0.090	-0.125*	0.124*	0.156**	0.113	0.085

GD_SSR_: genetic distance calculated based on SSR marker; GD_SNP_: genetic distance calculated based on SNP marker. A, C, D, and E indicate the F_1_ population, respectively. PH, plant height; BW, boll weight; LP, lint percentage; BN, boll number; FL, fiber length; FS, fiber strength; MIC, micronaire; FU, fiber uniformity; FE, fiber elongation rate; SCI, spinning consistency index. *, ** Indicate significance at *P* < 0.05 and *P* < 0.01, respectively.

In summary, the overall analysis results of the correlation between GD_SSR_ and GD_SNP_ in the four populations was inconsistent, and the correlation of group E was stronger than that of other groups.

### Relationship between genetic distance and BPH

The correlation results between GD of SSR markers and BPH showed that the GD_SSR_ was negatively correlated with the BPH of LP, FL, FS, MIC, FU, and SCI but positively correlated with the BPH of PH (Table 4). From the correlation results between GD of SNP markers and BPH, we observed that the GD_SNP_ was negatively correlated with the BPH of LP, BN, FL, MIC, and FE, and positively correlated with the BPH of PH and BW (Table 4).

**Table 4:** Correlation coefficients (*r*) of genetic distance with best-parent heterosis (BPH) of yield and fiber quality-related traits based on SSR and SNP marker.

Trait	GD_SSR_				GD_SNP_			
	A	C	D	E	A	C	D	E
PH	-0.007	-0.050	0.026	0.234**	-0.051	-0.011	0.015	0.155**
BW	-0.059	-0.056	0.070	0.027	-0.041	-0.039	0.056	0.126*
LP	-0.076	-0.200**	-0.264**	-0.204**	-0.118*	-0.307**	-0.328**	-0.361**
BN	0.081	-0.149	-0.064	0.032	-0.056*	-0.136*	-0.057	0.050
FL	-0.101	-0.037	-0.037	-0.206**	-0.113	-0.040	-0.045	-0.121*
FS	-0.050	-0.061	-0.054	-0.217**	-0.070	-0.007	-0.022	0.036
MIC	-0.033	-0.210*	-0.073	-0.223**	-0.179**	-0.237**	-0.150*	-0.192**
FU	-0.009	0.066	0.030	-0.141*	0.003	0.058	0.032	0.004
FE	-0.071	-0.067	-0.009	0.074	-0.170*	0.082	-0.022	-0.012
SCI	-0.032	-0.036	-0.050	-0.201**	-0.046	-0.006	-0.054	-0.009

GD_SSR_: genetic distance calculated based on SSR marker; GD_SNP_: genetic distance calculated based on SNP marker. A, C, D, and E indicate the F_1_ population, respectively. PH, plant height; BW, boll weight; LP, lint percentage; BN, boll number; FL, fiber length; FS, fiber strength; MIC, micronaire; FU, fiber uniformity; FE, fiber elongation rate; SCI, spinning consistency index. *, ** Indicate significance at *P* < 0.05 and *P* < 0.01, respectively.

In summary, the overall analysis results of the correlation between GD_SSR_, GD_SNP_ and the BPH of ten traits were consistent. The overall results were consistent with the correlation trends of F_1_s performance, but the correlation was weak.

## Discussion

### Genetic distance between parents assessed by SSR and SNP markers

With the rapid development and spread of molecular marker technology, these molecular markers have been used widely in analyses of GD, genetic diversity, population structure, genetic mapping, and linkage mapping. Earlier at the end of the twentieth century, some studies have used RFLP and SSR markers to study the relationship between GD and heterosis, and proposed that the relationship between GD and heterosis could be predicted by genetic differences [17]. Subsequently, a number of studies used RAPD [18], AFLP [19, 20], SSR [21–23], EST-SSR [24, 25], insertion-deletion (InDel) [11] and SNP markers [13, 26–28] to study the relationship between GD and heterosis. Previous studies used different molecular marker types and those results were also different, but the GD was not compared. SSR markers amplify products of different lengths according to the different number of tandem repeats in the core sequences of different materials to obtain the different genotypes of the population. The tandem repeats are mainly distributed in the non-coding region. SNP markers represent the whole genomic information of target species. Compared to traditional SSR markers, SNP markers have good genome-wide coverage. In this study, both SSR and SNP markers were used to study the GD between parents. There was a significant positive correlation between these two GDs (*r* > 0.264, *P* < 0.05) and we found that the SNP marker was more accurate and efficient than SSR marker to study the relationship between GD and heterosis.

### Plant heterosis prediction based on genetic distance

In recent years, methods have been sought to allow initial selection of parents intended for heterosis crossing. Previous studies attempted to analyze the relationship between GD and heterosis have resulted in different conclusions in various species, including wheat, sesame, rapeseed, cacao, eggplant, maize, and pearl millet. Few studies have used GD to estimate F_1_ performance and heterosis for improving the breeding efficiency on cotton heterosis utilization. In this study, under the condition of grouping according to different male parents, the GD of the two molecular markers were significantly negatively correlated with F_1_s performance and BPH of LP and MIC. The correlation between GD and MPH of each trait was weak. The correlation between GD_SNP_ and F_1_s performance, MPH and BPH was stronger than that of GD_SSR_. In addition, according to the clustering result by GD based on SNP, we found that all the F_1_s could be divided into five groups, and its average values of GD was 0.295, 0.287, 0.277, 0.275, and 0.261 for Group I, III, IV, V and II, respectively (Fig. 1). Meanwhile, we found that the lint percentage of Group I and III was significantly lower than Group IV, V and II (Fig. 3). Group II, which had the lowest GD, showed more bigger values in lint percentage, micronaire, fiber length, and fiber elongation ratio than Group I and III. All these results indicated that genetic distance between parents can be a valuable indicator for heterosis predication, especially for lint percentage micronaire, fiber length, and fiber elongation ratio and F_1_ crosses clustered in Group II had more commercial values in hybrid cotton breeding.

Positive correlations between GD and heterosis were reported in maize, wheat, pearl millet, *Brassica napus*, *Brassica oleracea*, cacao, and rapeseed. In maize, the GD between parental components, as determined by the SNP and SilicoDArT markers was significantly correlated with the heterosis effect observed in the majority of the yield structure features, as well as the yield itself [12]. Nie et al. reported a significant correlation between GD and MPH of 1000-grain weight in wheat [13]. In pearl millet, moderate positive significant correlations were found between GD and MPH for grain yield (*r* = 0.37, *p* < 0.01) and BPH for grain yield (*r* = 0.33, *p* < 0.01), respectively [21]. Nikzad et al. found a positive correlation for the genetic distance of the inbred lines from the common *Brassica napus* parent with MPH for seed yield (*r* = 0.31) and hybrid yield (*r* = 0.26) [23]. Significant correlation was observed between GD and MPH of plant height, gross plant weight, net curd weight, leaf width, curd diameter and total marketable yield in *Brassica oleracea* [25]. In cacao, a significant positive correlation of 0.39 was found between GD and SCA for yield [26]. Studies in rapeseed showed that GD evaluated by total molecular markers (GD_total_) had no correlation with heterosis but GD measured by favoring markers (GD_favor_) significantly and positively correlated with the number of seeds per silique, thousand seed weight, seed yield per plant and seed yield per plot for high-check heterosis and sum of parental general combining ability [29].

However, some investigations also showed no or weak correlation between GD and heterosis in wheat, pearl millet, sesame, eggplant, and maize. Nie et al. observed weak associations between the GD based on SNP and MPH or BPH of spikelet number, harvested spikes and yield in wheat [13]. Chen et al. found GD based on SSR markers poorly correlated with F_1_ performance, MPH and SCA in wheat [30]. Gupta et al. found that the GD was not correlated with heterosis of grain yield in pearl millet [21]. Pandey et al. revealed a weak association of GD with F_1_ performance in sesame [22]. In eggplant, GD assessed through SNPs showed a diminutive correlation with the hybrid means, heterosis, and SCA values [27]. In a previous study of maize lethal necrosis, a very low and negative correlation was observed between parental lines marker-based genetic distance and heterosis [31].

Betran et al. suggested that heterosis can be better predicted only when GD is smaller than a certain threshold [32]. Moreover, studies have suggested that the correlation is dependent on the investigated germplasm and GD calculation methods [33]. Previous studies showed that the efficiency of predicting heterosis by GD estimates was improved by selecting markers tightly linked to the QTL affecting heterosis of the target trait [34]. This suggested that higher heterosis was not from crosses between parents with largest GD, but mainly from those with intermediate GD. Significance of molecular marker-based GD in prediction of heterosis inevitably depends upon the methods used to calculate GD, molecular marker types, genome coverage of molecular marker, genome region of molecular marker, types of germplasm, breeding system, traits under consideration, and environmental conditions.

In this study, the low and insignificant correlation in certain traits may be due to the inadequate genome coverage, lack of association between markers and trait-controlling genes and epistasis among the quantitative trait loci.

## Conclusions

In this study, we used both SSR and SNP markers to estimate the GD between parents and to investigate the efficiency of the prediction of hybrid performance based on GD. Our study found that all the female parents could be divided into five groups based on GD_SNP_ cluster result and the F_1_ performance between these five groups showed significant differences in LP, MIC, FS, and FE. Furthermore, the correlation between GD and F_1_ performance, MPH and BPH were significant negative for lint percentage and micronaire. Overall, our results suggested that GD between parents could be helpful in heterosis prediction for LP and MIC and will be beneficial for heterotic group categorization and parental selection in hybrid cotton breeding.

## Methods

### Plant material

A total of 286 Upland cotton cultivars and lines were selected as parents in this study. All the accessions were collected from different ecological regions in China and from 13 different countries, represented a wide range of genetic backgrounds. The accessions used in this study were 136 elite cultivars, 103 historical cultivars (cultivated before 2000) and 47 exotic cultivars from 13 different countries. Among them, four elite cultivars (Zhong7886, A971, 4133, and SGK9708) with excellent comprehensive characters in China were selected as male parents. All the seeds were stored in the National Germplasm Mid-term Bank of the Institute of Cotton Research (ICR), the Chinese Academy of Agricultural Sciences (CAAS). The detailed information of 286 accessions is listed in Table S2.

### Field trial

The field experiments were conducted at the Yellow River region and Yangtze River region during 2012–2013 growing season. 1128 F_1_s were divided into four groups (A, C, D, E) according to their male parents. All 286 parents, F_1_s, and three control cultivars (Lumianyan 28, Ruiza 816 and Ezamian 10) were planted at two different experimental sites in two years. The four groups of locations in the same year were all in the same cotton region of China (Yangtze River valley or Yellow River valley). The experiment was conducted in a randomized complete block design, plots consisted of three rows each, 8 m long with a row spacing of 0.25 m. The field management was carried out according to the routine operation of local field production.

### Character investigation and data collection

A total of ten yield and fiber quality traits were collected from the middle row of each plot. One week after topping, plant height (PH, cm) was measured from the ground level to the tip in ten randomly selected plants. After attaining 70% of boll opening, ten mature bolls were randomly selected to investigate the boll number per plant (BN, No.) for each plot. After harvesting, boll weight (BW, g) and lint percentage (LP, %) were calculated by 30 bolls. Fiber quality traits including fiber length (FL, mm), fiber strength (FS, cN/tex), fiber length uniformity (FU, %), fiber elongation (FE, %), spinning consistency index (SCI, %) and micronaire (MIC) were determined by Cotton quality Supervision and Inspection Center of China Agriculture and Village Ministry (Anyang).

### Evaluation of heterosis

The mid-parent heterosis (MPH) and better-parent heterosis (BPH) were calculated by the formulas as follows: MPH = [F_1_-(P_1_ + P2_)_/2]/[(P_1_ + P_2_)/2] × 100% and BPH = (F_1_-HP)/HP × 100%, where F_1_ is the value of F_1_ hybrids, P_1_ and P_2_ are the phenotypic value of parents, HP is the phenotypic value of higher value parents.

### DNA extraction and genotyping

The fresh leaves of 286 parents were collected in the field, and the genomic DNA was extracted by CTAB method [35]. The concentration and purity of DNA were determined by Nano Drop2000 spectrophotometer, and the quality was determined by 1% agarose gel electrophoresis. A total of 198 polymorphic SSR markers were utilized from previous studies and listed in Table S3 [36].

The DNA concentration of qualified samples were adjusted to 100 ng/μL for restriction-site associated DNA sequencing (RAD-Seq) by Huada Gene Co., Ltd. (Shenzhen, China). The steps were as follows: (1) DNA digestion; (2) add bar-coded adapters; (3) DNA fragmentation; (4) DNA recovery and purification; (5) DNA amplification; (6) DNA recovery and purification; (7) sequenced on Illumina Hiseq 2000 system. The raw reads were aligned with *G.hirsutum* L. TM-1 reference genome v 1.1 (http://mascotton.njau.edu.cn/info/1054/1118.htm) by BWA software and the parameters were set to mem-t8. SNP genotypic data were obtained by SNP Calling, with GATK and SAMTools packages [37, 38]. The probability of the fragments mapped to the reference genome was 93.4–99.6%, the coverage on the genome was 0.07–7%, and the average sequencing depth was 1.48. The sequencing data had been deposited to NCBI under the accession number: PRJNA353524.

### Evaluation of genetic distance and correlation analysis

The genetic distance (GD) of SSR markers between parents was determined according to Nei’s et al. by Powermarker 3.25 [39, 40]. The formula is GD_SSR_ = 1-2N_ab_/(N_a_ + N_b_), where N_ab_ represent the SSR marker numbers amplified in both sample a and b, and N_a_ and N_b_ represent amplified SSR marker numbers in sample a and b, respectively. The GD of SNP markers between parents were calculated by TASSEL 5.0 based on the identity-by-state (IBS) genetic distance as GD_SNP_ = 1-IBS [41]. The average performance of MPH and BPH of ten yield and fiber quality related traits of 1128 F_1_s were analyzed by Graphpad prism 7.0. The packages ggplot2 and GGally in R software were used to analyze the correlation between GD of SSR and SNP marker and F_1_s performance, MPH, and BPH. Pearson’s correlation coefficients (*r*) were used to analyze the correlation between parent traits and F_1_s performance, MPH, and BPH and tested at *P* = 0.05 and 0.01.

## Supplementary Information

**Additional file 1 Table S1** Genetic distance matrix between parents assessed with SSR and SNP markers.

**Additional file 2 Table S2** The list of 286 Upland cotton (*Gossypium hirsutum* L.) parents and their origin and classification

**Additional file 3 Table S3** 198 polymorphic SSR markers amplified in 286 parents.

**Additional file 4 Fig. S1** The heatmap shows the number of SNP per 500 kb on the chromosome. The darker the color (red), the higher the density, and the number after each chromosome represents the total number of SNP on that chromosome.

**Additional file 5 Fig. S2** Clustering of 1128 F_1_s into three groups using genetic distance based on SSR markers. A, Clustering result by genetic distance based on SSR markers. B, The composition of SNP clustering groups in three SSR clustering groups.

**Additional file 6 Fig. S3** Boxplots showing the distribution of genetic distance for the Elite×Elite, Exotic×Elite, and Historic×Elite hybrids.

## Data Availability

The SNP data generated during the current study are available in NCBI (accession number: PRJNA353524). The SSR data can be found in additional files. The phenotypic datasets during the current study are available from the corresponding author on reasonable request. The *G.hirsutum* L. TM-1 reference genome can be downloaded from the website: http://mascotton.njau.edu.cn/info/1054/1118.htm.

## Abbreviations

BPH: Best parent heterosis
BN: Boll number
BW: Boll weight
FE: Fiber elongation rate
FL: Fiber length
FS: Fiber strength
FU: Fiber uniformity
GD: Genetic distance
InDel: insertion-deletion
LP: Lint percentage
MIC: Micronaire
MPH: Mid-parent heterosis
PH: Plant height
RAD-seq: restriction-site associated DNA sequencing
RAPD: Randomly amplified polymorphic DNA
RFLP: Restriction fragment length polymorphism
SCA: Specific combining ability
SCI: Spinning consistent index
SNP: Single nucleotide polymorphism
SSR: Simple sequence repeat SSR

## References

[1] Chen ZJ, Scheffler BE, Dennis E, Triplett BA, Zhang T, Guo W, Chen X, Stelly DM, Rabinowicz PD, Town CD (2007). Toward sequencing cotton (*Gossypium*) genomes. Plant Physiol.

[2] Shull GH. The composition of a field of maize. J Heredity. 1908, os-4(1):296–301.

[3] Tang F, Xiao W (2013). Genetic effects and heterosis of within-boll yield components in upland cotton (*Gossypium hirsutum* L.). Euphytica..

[4] Gowda M, Kling C, Würschum T, Liu W, Maurer HP, Hahn V, Reif JC (2010). Hybrid breeding in durum wheat: heterosis and combining ability. Crop Sci.

[5] Reif JC, Zhao Y, Würschum T, Gowda M, Hahn V, Léon J (2013). Genomic prediction of sunflower hybrid performance. Plant Breed.

[6] Beyene Y, Gowda M, Suresh LM, Mugo S, Olsen M, Oikeh SO, Juma C, Tarekegne A, Prasanna BM (2017). Genetic analysis of tropical maize inbred lines for resistance to maize lethal necrosis disease. Euphytica..

[7] Kumar Soni S, Tiwari S, Newmah TJ, Dossou Aminon I, Sundaram RM (2018). Prediction of hybrid performance in crop plants: molecular and recent approaches. Int J Curr Microbiol Appl Sci.

[8] Hale AL, Farnham MW, Nzaramba MN, Kimbeng CA (2007). Heterosis for horticultural traits in broccoli. Theor Appl Genet.

[9] Larièpe A, Moreau L, Laborde J, Bauland C, Mezmouk S, Décousset L, Mary-Huard T, Fiévet JB, Gallais A, Dubreuil P (2017). General and specific combining abilities in a maize (*Zea mays* L.) test-cross hybrid panel: relative importance of population structure and genetic divergence between parents. Theor Appl Genet.

[10] Fujimoto R, Uezono K, Ishikura S, Osabe K, Peacock WJ, Dennis ES (2018). Recent research on the mechanism of heterosis is important for crop and vegetable breeding systems. Breed Sci.

[11] Hu Y, Mao B, Peng Y, Sun Y, Pan Y, Xia Y, Sheng X, Li Y, Tang L, Yuan L (2014). Deep re-sequencing of a widely used maintainer line of hybrid rice for discovery of DNA polymorphisms and evaluation of genetic diversity. Mol Gen Genomics.

[12] Tomkowiak A, Bocianowski J, Radzikowska D, Kowalczewski PL (2019). Selection of parental material to maximize heterosis using SNP and SilicoDarT markers in maize. Plants..

[13] Nie Y, Ji W, Ma S (2019). Assessment of heterosis based on genetic distance estimated using SNP in common wheat. Agronomy..

[14] Zhang XQ, Wang XD, Jiang PD, Hua SJ, Zhang HP, Dutt Y (2007). Relationship between molecular marker heterozygosity and hybrid performance in intra- and interspecific hybrids of cotton. Plant Breed.

[15] Zeng L, Meredith WR (2011). Relationship between SSR-based genetic distance and cotton F_2_ hybrid performance for lint yield and fiber properties. Crop Sci.

[16] SSP YAA, Manjula SM, Nadaf HL, Patil BC (2013). Relationship between SSR-based molecular marker and cotton F_1_ inter specific hybrids performance for seed cotton yield and fiber properties. Genomics Appl Biol.

[17] Zhang Q, Gao YJ, Yang SH, Ragab RA, Maroof MA, Li ZB (1994). A diallel analysis of heterosis in elite hybrid rice based on RFLPs and microsatellites. Theor Appl Genet.

[18] Teklewold A, Becker HC (2006). Comparison of phenotypic and molecular distances to predict heterosis and F_1_ performance in Ethiopian mustard (*Brassica carinata* a. Braun). Theor Appl Genet.

[19] Frisch M, Thiemann A, Fu J, Schrag TA, Scholten S, Melchinger AE (2010). Transcriptome-based distance measures for grouping of germplasm and prediction of hybrid performance in maize. Theor Appl Genet.

[20] Schrag TA, Mohring J, Melchinger AE, Kusterer B, Dhillon BS, Piepho HP, Frisch M (2010). Prediction of hybrid performance in maize using molecular markers and joint analyses of hybrids and parental inbreds. Theor Appl Genet.

[21] Gupta SK, Nepolean T, Shaikh CG, Rai K, Hash CT, Das RR, Rathore A (2018). Phenotypic and molecular diversity-based prediction of heterosis in pearl millet (*Pennisetum glaucum* L. (R.) Br.). Crop J.

[22] Pandey SK, Dasgupta T, Rathore A, Vemula A (2018). Relationship of parental genetic distance with heterosis and specific combining ability in sesame (*Sesamum indicum* L.) based on phenotypic and molecular marker analysis. Biochem Genet.

[23] Nikzad A, Kebede B, Pinzon J, Bhavikkumar J, Wang X, Yang RC, Rahman H (2019). Potential of the C genome of the different variants of *Brassica oleracea* for heterosis in spring *B napus* canola. Front Plant Sci.

[24] Su J, Zhang F, Yang X, Feng Y, Yang X, Wu Y, Guan Z, Fang W, Chen F. Combining ability, heterosis, genetic distance and their intercorrelations for waterlogging tolerance traits in chrysanthemum. Euphytica. 2017, 213(2).

[25] Singh S, Dey SS, Bhatia R, Kumar R, Sharma K, Behera TK (2019). Heterosis and combining ability in cytoplasmic male sterile and doubled haploid based Brassica oleracea progenies and prediction of heterosis using microsatellites. PLoS One.

[26] Mustiga GM, Gezan SA, Phillips-Mora W, Arciniegas-Leal A, Mata-Quiros A, Motamayor JC. Phenotypic description of *Theobroma cacao* L. for yield and vigor traits from 34 hybrid families in Costa Rica based on the genetic basis of the parental population. Front Plant Sci. 2018, 9:808. 10.3389/fpls.2018.00808 PMC6018478 29971076

[27] Kaushik P (2019). Genetic analysis for fruit phenolics content, flesh color, and browning related traits in eggplant (*Solanum melongena*). Int J Mol Sci.

[28] Napolitano M, Terzaroli N, Kashyap S, Russi L, Jones-Evans E, Albertini E. Exploring heterosis in melon (*Cucumis melo* L.). Plants. 2020, 9(2):282. 10.3390/plants9020282 PMC7076541 32098173

[29] Tian HY, Channa SA, Hu SW. Relationships between genetic distance, combining ability and heterosis in rapeseed (*Brassica napus* L.). Euphytica. 2016, 213(1).

[30] Chen X, Sun D, Rong DF, Sun G, Peng J (2010). Relationship of genetic distance and hybrid performance in hybrids derived from a new photoperiod-thermo sensitive male sterile wheat line 337S. Euphytica..

[31] Nyaga C, Gowda M, Beyene Y, Murithi WT, Burgueno J, Toledo F, Makumbi D, Olsen MS, Das B, L MS et al. Hybrid breeding for MLN resistance: heterosis, combining ability, and hybrid prediction. Plants. 2020, 9:468. 10.3390/plants9040468 PMC7238107 32276322

[32] Betran FJ, Ribaut JM, Beck D, de Leon DG (2003). Genetic diversity, specific combining ability, and heterosis in tropical maize under stress and nonstress environments. Crop Sci.

[33] Melchinger AE, Lee M, Lamkey KR, Hallauer AR, Woodman WL (1990). Genetic diversity for restriction fragment length polymorphisms and heterosis for two diallel sets of maize inbreds. Theor Appl Genet.

[34] Charcosset A, Essioux L (1994). The effect of population structure on the relationship between heterosis and heterozygosity at marker loci. Theor Appl Genet.

[35] Paterson AH, Brubaker CL, Wendel JF (1993). A rapid method for extraction of cotton (*Gossypium Spp*.) genomic DNA suitable for RFLP or PCR analysis. Plant Mol Biol Rep.

[36] Ademe MS, He S, Pan Z, Sun J, Du X (2017). Association mapping analysis of fiber yield and quality traits in upland cotton (*Gossypium hirsutum* L.). Mol Gen Genomics.

[37] Li H, Handsaker B, Wysoker A, Fennell T, Ruan J, Homer N, Marth G, Abecasis G, Durbin R (2009). Genome project data processing S. the sequence alignment/map format and SAMtools. Bioinformatics..

[38] McKenna A, Hanna M, Banks E, Sivachenko A, Cibulskis K, Kernytsky A, Garimella K, Altshuler D, Gabriel S, Daly M (2010). The genome analysis toolkit: a MapReduce framework for analyzing next-generation DNA sequencing data. Genome Res.

[39] Nei M, Tajima F, Tateno Y (1983). Accuracy of estimated phylogenetic trees from molecular data. J Mol Evol.

[40] Liu K, Muse SV (2005). PowerMarker: an integrated analysis environment for genetic marker analysis. Bioinformatics..

[41] Bradbury PJ, Zhang Z, Kroon DE, Casstevens TM, Ramdoss Y, Buckler ES (2007). TASSEL: software for association mapping of complex traits in diverse samples. Bioinformatics..

