# Supplementary figures and images for: Assessment of heterosis based on parental genetic distance estimated with SSR and SNP markers in upland cotton (Gossypium hirsutum L.)
Source: BMC Genomics. 2021 Feb 18;22:123. doi: 10.1186/s12864-021-07431-6 (PMC7891138; doi:10.1186/s12864-021-07431-6)

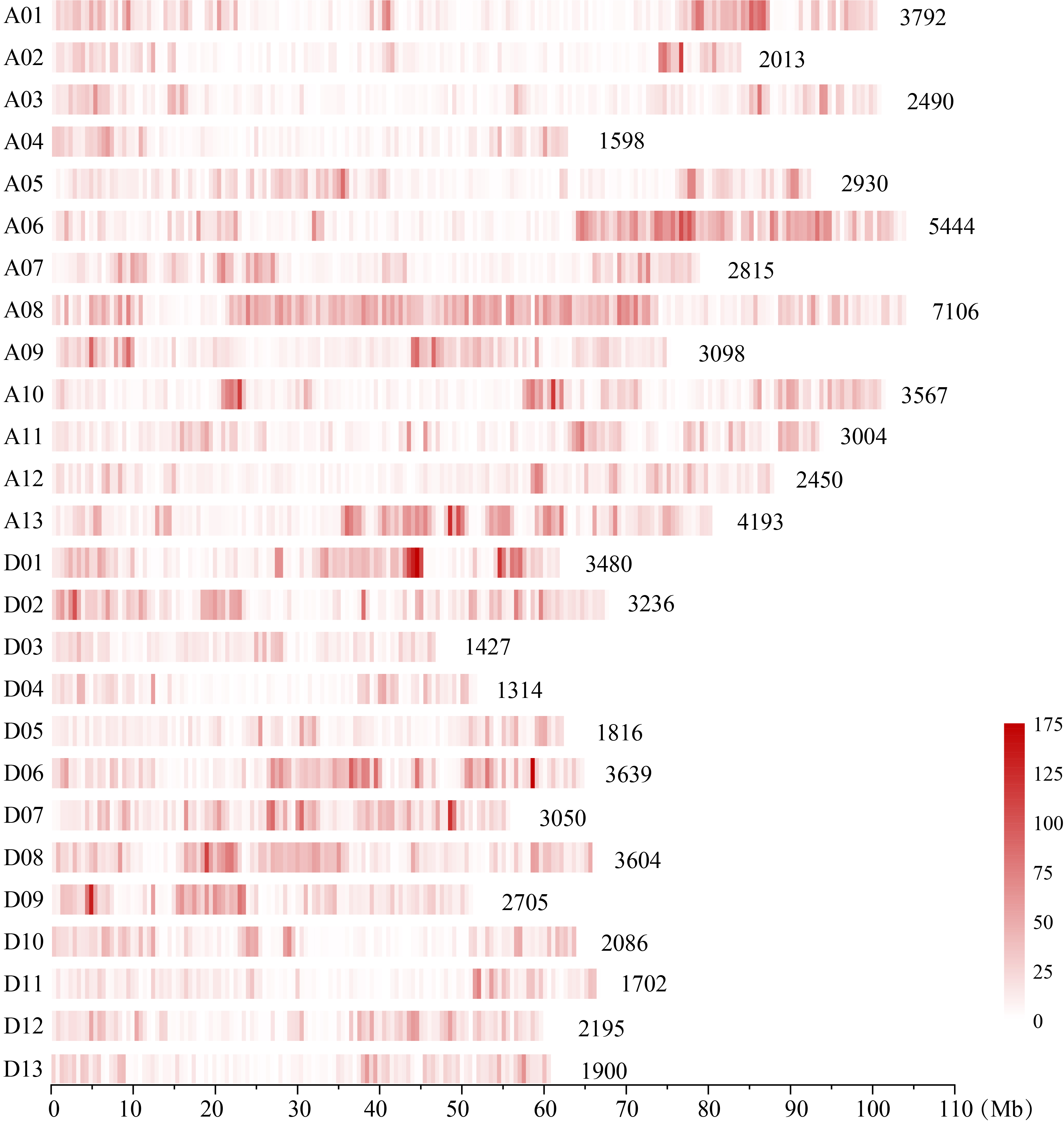

Supplement: Supplementary file 4 — Additional file 4 Fig. S1 The heatmap shows the number of SNP per 500 kb on the chromosome. The darker the color (red), the higher the density, and the number after each chromosome represents the total number of SNP on that chromosome. [file 12864_2021_7431_MOESM4_ESM.jpg]

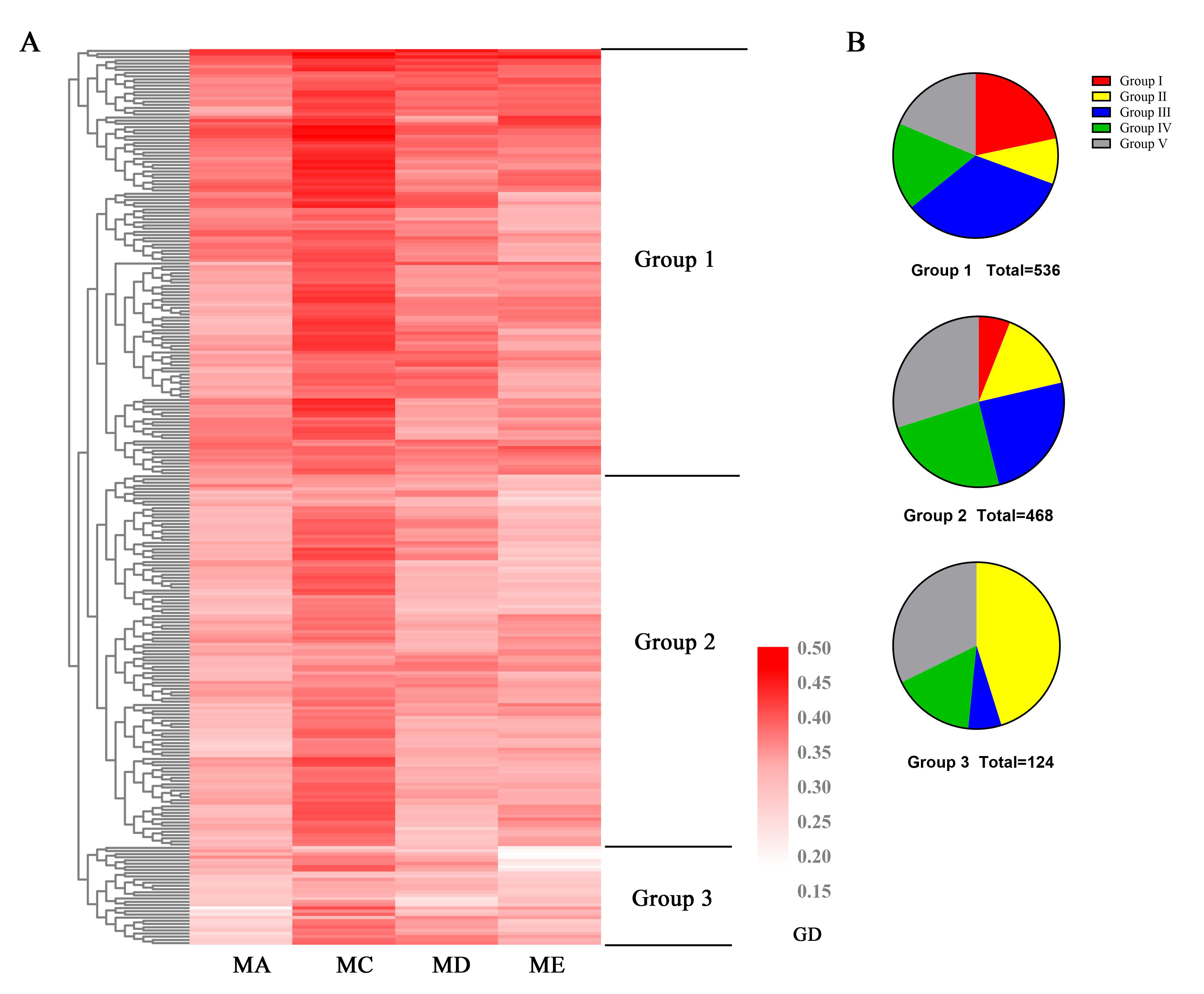

Supplement: Supplementary file 5 — Additional file 5 Fig. S2 Clustering of 1128 F1s into three groups using genetic distance based on SSR markers. A, Clustering result by genetic distance based on SSR markers. B, The composition of SNP clustering groups in three SSR clustering groups. [file 12864_2021_7431_MOESM5_ESM.jpg]

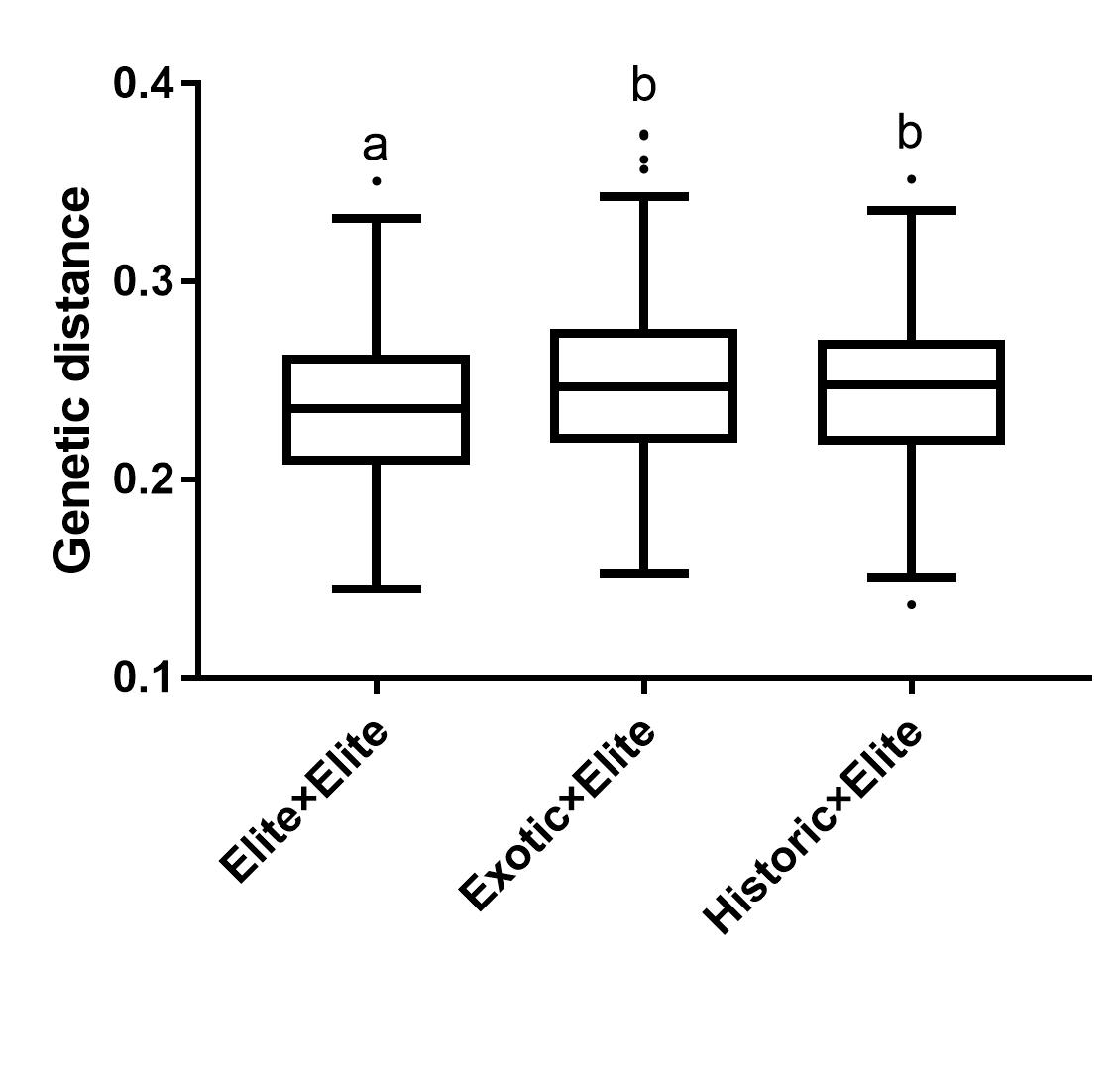

Supplement: Supplementary file 6 — Additional file 6 Fig. S3 Boxplots showing the distribution of genetic distance for the Elite×Elite, Exotic×Elite, and Historic×Elite hybrids. [file 12864_2021_7431_MOESM6_ESM.jpg]
